# Analysis of High-Altitude De-Acclimatization Syndrome after Exposure to High Altitudes: A Cluster-Randomized Controlled Trial

**DOI:** 10.1371/journal.pone.0062072

**Published:** 2013-05-01

**Authors:** Binfeng He, Jianchun Wang, Guisheng Qian, Mingdong Hu, Xinming Qu, Zhenghua Wei, Jin Li, Yan Chen, Huaping Chen, Qiquan Zhou, Guansong Wang

**Affiliations:** 1 Institute of Respiratory Diseases, The Second Affiliated Hospital of the Third Military Medical University, Chongqing, China; 2 Department of High Altitude Diseases, College of High Altitude Military Medicine, Third Military Medical University, Chongqing, China; Mayo Clinic College of Medicine, United States of America

## Abstract

The syndrome of high-altitude de-acclimatization commonly takes place after long-term exposure to high altitudes upon return to low altitudes. The syndrome severely affects the returnee's quality of life. However, little attention has been paid to careful characterization of the syndrome and their underlying mechanisms. Male subjects from Chongqing (n = 67, 180 m) and Kunming (n = 70, 1800 m) visited a high-altitude area (3650 m) about 6 months and then returned to low-altitude. After they came back, all subjects were evaluated for high-altitude de-acclimatization syndrome on the 3^rd^, 50^th^, and 100^th^. Symptom scores, routine blood and blood gas tests, and myocardial zymograms assay were used for observation their syndrome. The results showed that the incidence and severity of symptoms had decreased markedly on the 50^th^ and 100^th^ days, compared with the 3^rd^ day. The symptom scores and incidence of different symptoms were lower among subjects returning to Kunming than among those returning to Chongqing. On the 3^rd^ day, RBC, Hb, Hct, CK, CK-MB, and LDH values were significantly lower than values recorded at high altitudes, but they were higher than baseline values. On the 50^th^ day, these values were not different from baseline values, but LDH levels did not return to baseline until the 100^th^ day. These data show that, subjects who suffered high-altitude de-acclimatization syndrome, the recovery fully processes takes a long time (≥100^th^ days). The appearance of the syndrome is found to be related to the changes in RBC, Hb, Hct, CK, CK-MB, and LDH levels, which should be caused by reoxygenation after hypoxia.

## Introduction

High-altitude de-acclimatization is a multifaceted process involving the loss of high-altitude acclimation over time after an individual who has acclimated to high altitudes returns to lower altitudes [1]. The changes in physiological functions experienced by explorers returning to sea level from high altitudes were documented as early as 1908 [2]. High-altitude de-acclimatization has been noted in explorers [3], athletes [4], military personnel [5], high-altitude railway workers [6], and workers in high-altitude mines [7]. Hypoxia is always considered the main threat to mammals at these altitudes, so high-altitude de-acclimatization is also considered de-acclimatization to hypoxia [1]. Over the past few decades, many studies have shown that it involves not only hormone levels and the hematologic, respiratory, and cardiovascular systems but also the nervous system and psychology [2], [8]–[13].

Many studies of high-altitude de-acclimatization have been reported. However, the symptoms experienced by subjects have received little attention. Individuals subjected to short-term exposure to high altitudes, on the scale of a few days, experience symptoms so light that they cannot always be observed. However, some climbers complain of headaches and fidgetiness when they descend to sea level from high altitudes. Zubieta-Calleja showed that visitors from high altitudes suffered from excessive somnolence, lower limb edema, diminished reflexes, and inadequate fine motor coordination when they descend to sea level [14]. Cui et al. demonstrated that 70.76% of individuals, who live at high altitudes for 10–30 years, present a series of clinical symptoms including fatigue, headache, and sleepiness [15]. Our previous study showed that 84.36% of individuals who had lived in Tibet for 10–20 years presented a series of clinical symptoms including fatigue, sleepiness, insomnia, unresponsiveness, memory loss, fidgetiness, headache, throat pain or discomfort, coughing, expectoration, chest tightness, flustering, increased appetite, decreased appetite, constipation, diarrhea, abdominal distention, abdominal pain, lumbago, arthralgia, and some abnormal physiological parameters of the cardiovascular, hematological, and respiratory systems upon returning to lower altitudes [16]. These symptoms were not observed in healthy individuals who had not visited high altitudes. Other studies have shown that several of these symptoms can last many years in some severe cases, and 1‰ of subjects experienced such severe symptoms that they had to return to high altitudes [17]. We refer to all of the above pathological features as a high-altitude de-acclimatization syndrome [18], [19].

Previous studies of high-altitude de-acclimatization syndrome have focused on long-term (>1 year) exposure to high altitudes. We have proposed that existing diagnostic and scoring criteria for high-altitude de-acclimatization are appropriate for populations subjected to long-term exposures to high altitudes [16], [20]. The incidences of symptoms, duration of symptoms, hematologic function, cardiovascular function, and levels of reactive oxygen species (ROS) associated with high-altitude de-acclimatization symptoms have been reported [16], [21]–[23].

Following the expansion of railway and air travel in Qinghai and Tibet, many people have come to live in high-altitude areas for relatively short periods of time (≤1 year) for such purposes as earthquake relief, commerce, education, and work. The incidence of de-acclimatization symptoms upon the return to lower altitudes has increased by as much as 100% [23]. Accurate understanding and treatment of this condition is hindered by diagnostic and scoring criteria designed for use with individuals subjected to long-term exposure to high altitudes, rather than fixed time exposure (≤1 year, >8 weeks). For this reason, we analyzed epidemiological study data from subjects who had returned to Chongqing, Kunming, and other areas after spending less than 1 year in high-altitude areas. We recommend modifications to the diagnostic and scoring criteria used with subjects exposed to high altitudes for fixed periods of time.

This report provides symptom scores and diagnostic criteria for fixed time (≤1 year, >8 weeks) de-acclimatization syndrome and demonstrates the symptoms generated are connected with the changes of red blood cell mass (RBC), hemoglobin (Hb), hematocrit (Hct), arterial partial pressure of oxygen (PaO2), partial pressure of carbon dioxide (PaCO2), oxygen saturation (SaO2), creatine kinase (CK), creatine kinase–MB (CK-MB), and lactate dehydrogenase (LDH), which have specific relationships with reoxygenation after hypoxia. The symptom scores were associated with differences in altitude, symptom characteristics, hematological cell levels, blood gas, and myocardial enzyme levels. They slowly returned to baseline, as indicated by measurements taken on the 3rd, 50th, and 100th days after the subjects' return to either Chongqing or Kunming. However, the recovery of fixed time de-acclimatization symptoms required a long time, more than 100 days.

## Methods

### Subjects and general protocols

Sixty-seven healthy men (33.42±8.12 years old) from Chongqing (180 m) and 70 healthy men (32.9±7.32 years old) from Kunming (1800 m) served as the high-altitude de-acclimatization syndrome group (HDAS group). Ninety-two healthy men (31.33±7.47 years old) from Chongqing and 87 healthy men from Kunming (30.33±11.59 years old) who had not been exposed to high altitudes served as controls. The two groups were not statistically different in age and place of permanent residence (P>0.05).

Subjects who came from Chongqing or Kunming maintained the same activity levels in Lhasa as in Chongqing or Kunming. (1) Altitude. Individuals arrived at the same altitude (3650 m) and worked at two spring water factories, respectively. (2) Working conditions. They worked in workshops. These were similar to workshops in Chongqing and Kunming with respect to work environment and temperature. (3) Work intensity. The subjects maintained the same level of intensity at all three locations. (4) Work day. The work day lasted for 7 hours per day, 5 days per week. Subjects kept the same schedule at high and low altitude. (5) Dietary pattern. The subjects kept the similar dietary pattern at all three locations. Subjects were required to eat three meals every day in their factory cafeteria. (6) Work season. All subjects worked in Lhasa from March to October or November. The weather during this period, summer and autumn, is suitable for this type of work, because oxygen content in air is higher (10%) than in winter (6%).

Physicians were trained by the trial manager to evaluate symptoms of subjects, and experienced technicians were trained standard procedures for collecting arterial blood before trail carried out. Subjects had been diagnosed with high-altitude de-acclimatization syndrome according to relevant diagnostic and scoring criteria. Symptom scores were assessed as the described below.

Subjects had worked in Lhasa (3650 m) about 6 months and then returned to Chongqing or Kunming by airplane. After they came back, all subjects were evaluated for high-altitude de-acclimatization syndrome on the 3^rd^, 50^th^, and 100^th^ days. Symptom scores, routine blood and blood gas tests, and myocardial zymograms assay were used for observation their syndrome. All Subjects provided written informed consent. This study was approved by the medical ethical committee of the Second Affiliated Hospital, Third Military Medical University.

### Diagnostic and scoring criteria for fixed period high-altitude de-acclimatization syndrome

The diagnostic and scoring criteria for high altitude de-acclimatization syndrome are based on epidemiological study data. They are modified versions of our previous scoring criteria [16].

#### 1. Essential diagnostic criteria for high altitude de-acclimatization syndrome

(1) Adult≤60 years old. (2) Recent return to lower altitude from a higher altitude. (3) Three or more of the following symptoms: fatigue, sleepiness, insomnia, unresponsiveness, memory loss, fidgety, headache, throat pain or discomfort, coughing, expectoration, chest tightness, flustering, increased appetite, decreased appetite, expectoration, diarrhea, abdominal distention, abdominal pain, lumbago, or arthralgia. (4) No significant relief of symptoms after 3 days of simple medication administered after the return to lower altitudes.

#### 2. Auxiliary diagnostic criteria for high altitude de-acclimatization syndrome

(1) Blood routine: RBC, Hb, and Hct levels high above baseline. (2) Myocardial enzymes: CK-MB and LDH levels above those of individual's native to low altitudes. (3) Urine: Microalbuminuria above that of individual's native to low altitudes. (4) Heart function: Pulmonary arterial pressure slightly higher than that of individuals native to high altitudes accompanied by left or right ventricular systolic and diastolic dysfunction. Raised Tei index coupled with low values of left ventricular ejection fraction (LVEF), right ventricular ejection fraction (RVEF), left ventricular fractional shortening (LVFS), and right ventricular fractional shortening (RVFS). (5) Brain function: Dysfunction of immediate short-term memory. (6) Hepatic function: Total bilirubin, ALT, and AST levels higher than those of individual's native to low altitudes.

Diagnosis of high altitude de-acclimatization included essential conditions and one Auxiliary condition.

#### 3. Exclusion criteria

(1) Symptoms directly attributable to primary diseases affecting the cardiovascular, respiratory, nervous, urinary, and hematological systems. (2) Cancer or leukemia. (3) Any history of highland heart disease or high-altitude polycythemia. (4) Recent history of flu, upper respiratory tract infection, infectious diarrhea, or similar symptoms.

#### 4. Classification and scoring criteria of the symptoms of high-altitude de- acclimatization syndrome (Tables 1 and 2)

**Table 1:** Classification and scoring criteria of the symptoms of high-altitude de-acclimatization syndrome.

Classification	Judgment standards	Scoring standards
±	Mild symptoms with no impact on daily life	0
+	Mild symptoms with slight impact on daily life, ameliorated after drug regimen	1
++	Severe symptoms affect daily life, somewhat alleviated after drug regimen	2
+++	Severe symptoms affect daily life, no significant relief after drug regimen	3

**Table 2:** Grading of high-altitude altitude de-acclimatization syndrome.

Classification	Diagnostic criteria
Almost no reaction (±)	Suspected symptoms (±) or 0–5 total points
Mild reaction (+)	Slight symptoms (+) or 6–15 total points
Moderate reaction (++)	More serious symptoms (++) or 16–25 total points
Severe reaction (+++)	Very serious symptoms (+++) or 26 or more total points

#### 5. Symptom scores

Symptom scores were evaluated according to the scoring criteria for fixed-duration high-altitude de-acclimatization syndrome.

### Biological measurements

#### 1. Blood gas assay

Technician is to follow physician order for collecting the sample of arterial blood gas. If sample collection was failed, all of blood gas data of this subject was been eliminated.

Blood gas collecting procedures: The skin over the puncture site in radial artery is cleaned with 70% isopropyl alcohol. Insert the needle through the skin into the artery taking care not to puncture the posterior wall of the artery. Withdraw the needle when an adequate sample has been obtained. Immediately place dry gauze over the puncture site and apply pressure. Maintain pressure over puncture site for a minimum of 5 minutes. All samples were analyzed within 30 min of arterial puncture using a blood gas analyzer (Lexington, MA, IL-1620, US).

#### 2. Collection and analysis of blood samples

Morning fasting venous blood (1 ml) was collected (with EDTA-K2), and samples were assayed within 2 hours using an hematology analyzer (Sysmex XE-2100, Japan) at the Forty-eighth Hospital in Lhasa, Xinqiao Hospital in Chongqing, and the 478th Hospital in Kunming. The operation was performed in strict according with the standard SOP. All equipment was used in accordance with the manufacturers' protocols. The same types of equipment were used at all three sites and they showed statistically similar levels of efficiency (P<0.01).

#### 3. Measurement of myocardial enzymes

Morning fasting venous blood (3 ml) was collected, centrifuged at 4000 r/min for 10 min to separate serum, and stored at −80°C before assay. The concentrations of CK, CK-MB, and LDH were measured at Xinqiao Hospital on a fully automated biochemistry analyzer (Olympus Au2700, Japan).

#### 4. Rates of change

Altitude de-acclimatization symptom scores recorded on the 3rd day after the return to plains areas served as baseline. Rate of change rate 1 = (score on 50th day-score on 3rd day)/score on 3rd day×100%; rate of change 2 = (score on 100th day-score on 3rd day)/score on 3rd day×100%.

RBC levels recorded at high altitudes served as a baseline value. Rate of change 1 = (value on 3rd day-value at high altitudes)/value at high altitudes×100%; rate of change 2 = (value on 50th day-value at high altitudes)/value at high altitudes×100%; rate of change 3 = (value on 100th day-value at high altitudes)/value at high altitudes×100%. The rate of change rate of CK, CK-MB, and LDH was same the same as the rate of change in RBC count.

### Statistical analysis

SPSS 15.0 for windows was used for statistical analysis. All data are presented as the arithmetic mean values (SD). Percent of symptoms dates ware compared by χ2 test. Symptoms score, RBC, Hb, Hct, MCV, MCH, MCHC, WBC, Neu%, CK, CK-MB and LDH obtained in the various sessions were compared by repeated- measure analysis of variance (ANOVA). The unpaired t-test was used as a post-hoc test to evaluate the significance of differences between Chongqing group and Kunming group. The level of significance was taken at P<0.05.

## Results

### Symptom scores

**Figure 1:**
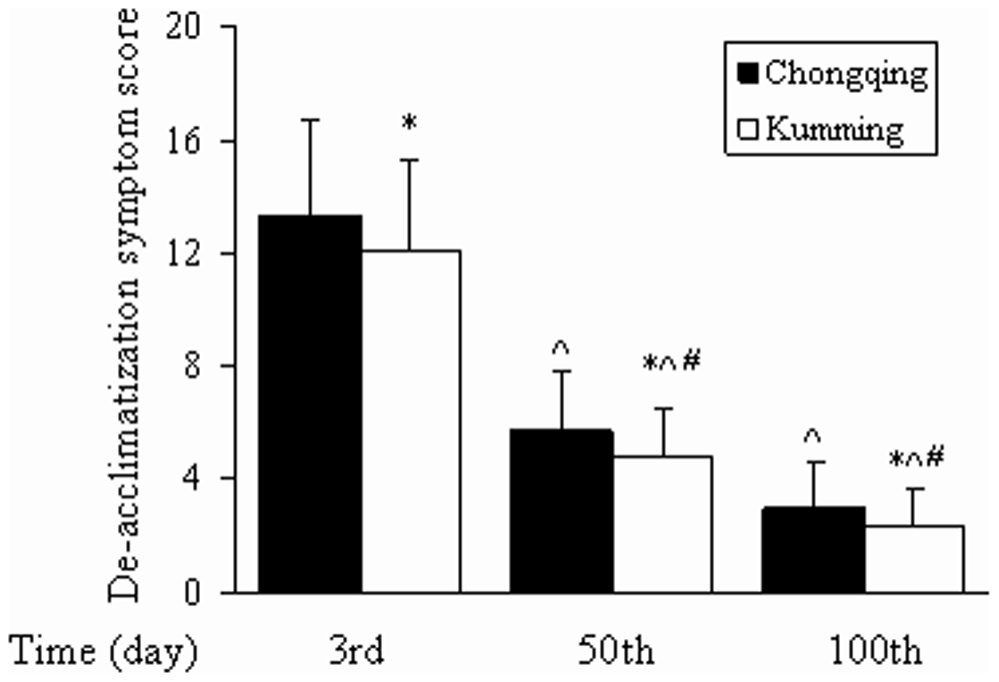
Symptom scores. Data are presented as mean ± SD. *P<0.05, relative to the Chongqing group. ^*P*<0.05, relative to the 3^rd^ day, ^#^ *P*<0.05, relative to the 50^th^ day.

The altitude de-acclimatization syndrome symptom scores of the Chongqing and Kunming groups were evaluated by physicians on the 3^rd^, 50^th^, and 100^th^ days upon returned to low-altitude areas after 6 months of exposure to high altitudes, as shown in Figure 1.

In the Chongqing group, the altitude de-acclimatization syndrome symptom scores of subjects were 13.38±3.31 on the 3^rd^ day, 5.71±2.04 on the 50^th^ day, and 3.05±1.51 on the 100^th^ day. Scores were significantly higher on the 3^rd^ day than on the 50^th^ or 100^th^ (both *P*<0.05). Scores were significantly higher on the 50^th^ day than on the 100^th^ (*P*<0.05).

In the Kunming group, altitude de-acclimatization syndrome symptom scores were 12.12±3.16 on the 3^rd^ day, 4.75±1.73 on the 50^th^ day, and 2.35±1.33 on the 100^th^ day. Scores were significantly higher on the 3^rd^ day than on the 50^th^ day or 100^th^ day (both *P*<0.05). Scores were significantly higher on the 50^th^ day than on the 100^th^ (*P*<0.05).

The Chongqing and Kunming groups were compared on the 3^rd^, 50^th^, and 100^th^ days after return to low-altitude areas. Altitude de-acclimatization syndrome symptom scores were significantly higher in the Chongqing group at all three points in time (all *P*<0.05).

These results indicate that the symptoms of altitude de-acclimatization syndrome should be improved over time. The symptoms were more serious in the Chongqing group than in the Kunming group.

**Figure 2:**
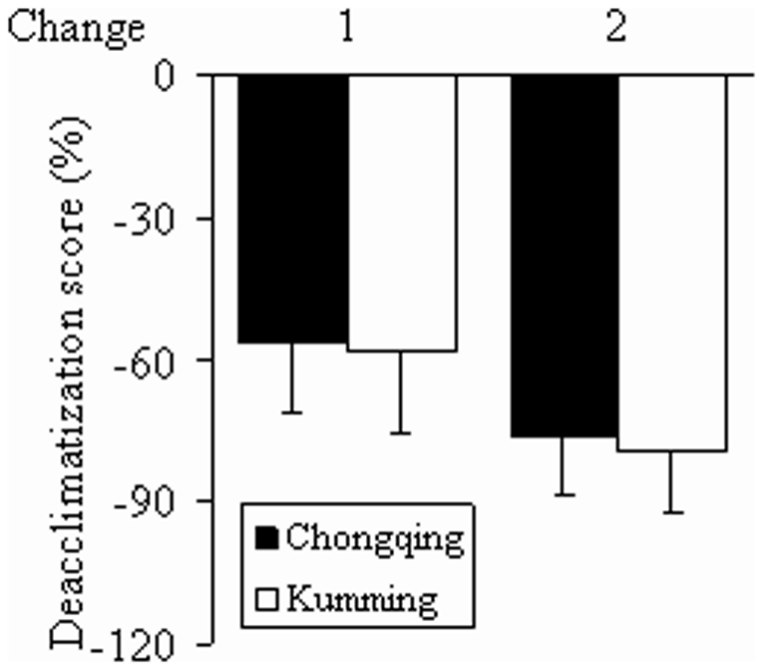
Rates of change in symptom scores. Data are presented as mean ± SD. Symptom scores recorded on the 3^rd^ day after return to low-altitude areas served as baseline values. Rate of change 1 = (score on 50^th^ day-score on 3^rd^ day)/score on 3^rd^ day×100%; rate of change 2 = (score on 100^th^ day-score on 3^rd^ day)/score on 3^rd^ day×100%. Rates of change 1 and 2 showed no significant difference between the Chongqing and Kunming groups (*P*>0.05).

Rates of change 1 and 2 showed no significant differences between the Chongqing group and Kunming group (both *P*>0.05) (Figure 2).

### Incidence

The incidence of symptoms of high altitude de-acclimatization syndrome in both Chongqing and Kunming subjects are illustrated in detail in Table 3.

**Table 3:** Incidence of symptoms of subjects after returning to Chongqing or Kunming after visiting high altitudes.

	Chongqing group (n = 67)			Kunming group (n = 70)		
Symptoms	3^rd^ day	50^th^ day	100^th^ day	3^rd^ day	50^th^ day	100^th^ day
Fatigue	49 (73.13%)	27 (40.29%)^#^	8 (11.94%)^&^	35 (50.00%)	19 (27.14%)^#^	6 (8.57%)^&^
Sleepiness	48 (71.64%)^*^	22 (32.83%)^#^	9 (13.43%)^&^	33 (47.14%)	20 (28.57%)^#^	11 (15.71%)
Insomnia	29 (43.28%)^*^	23 (34.32%)^*^	9 (13.43%)^&^	19 (27.14%)	13 (18.57%)	8 (11.42%)
Unresponsiveness	31 (46.26%)^*^	10 (14.92%)^*#^	5 (7.46%)	11 (15.71%)	8 (11.42%)	3 (4.28%)
Memory loss	31 (46.26%)^*^	20 (29.85%)	10 (14.92%)^&^	22 (31.42%)	18 (25.71%)	9 (12.85%)^&^
Agitation	30 (44.77%)^*^	24 (35.82%)	12 (17.91%)^&^	20 (28.57%)	12 (17.14%)	7 (10.00%)
Headache	36 (53.73%)^*^	27 (40.29%)^*^	14 (20.89%)^*&^	20 (28.57%)	12 (17.14%)	6 (8.57%)
Dizziness	49 (73.13%)^*^	27 (40.29%)^#^	23 (17.91%)	35 (50.00%)	26 (37.14%)	16 (22.85%)
Throat pain or discomfort	28 (41.79%)^*^	14 (20.89%)^#^	7 (10.44%)	15 (21.42%)	8 (11.42%)	4 (5.71%)^&^
Coughing	49 (73.13%)^*^	30 (44.76%)^*#^	21 (31.34%)^*^	37 (52.85%)	18 (14.28%)^#^	6 (8.57%)
Expectoration	31 (46.26%)	16 (23.88%)^#^	8 (26.86%)	35 (50.00%)	8 (11.42%)^#^	5 (7.14%)
Chest tightness	37 (55.22%)^*^	23 (34.32%)^*#^	10 (14.92%)^&^	17 (24.28%)	10 (14.28%)	6 (8.57%)
Flustering	41 (61.19%)^*^	25 (37.31%)^*#^	18 (26.86%)^*^	25 (35.71%)	13 (18.57%)^#^	5 (7.14%)^&^
Increased appetite	31 (46.26%)	11 (16.41%)^#^	3(4.47%)^&^	22 (31.42%)	8 (11.42%)^#^	1 (1.42%)^&^
Decreased appetite	27 (40.29%)^*^	17 (25.37%)^*#^	5 (7.46%)^&^	17 (24.28%)	10 (14.28%)	4 (5.71%)
Constipation	22 (32.83%)	8 (11.94%)^#^	1 (1.49%)^&^	19 (27.14%)	6 (8.57%)	0 (0.00%)^&^
Diarrhea	18 (26.86%)	8 (11.94%)^#^	1 (1.49%)^&^	13 (18.57%)	6 (8.57%)	1 (1.42%)
Abdominal distention	44 (66.67%)^*^	28 (41.79%)	20 (29.85%)^&^	32 (45.71%)	20 (28.57%)^#^	6 (8.57%)^&^
Abdominal pain	40 (59.70%)	29 (43.28%)^#^	13 (19.40%)	31 (44.28%)	21 (30.00%)	9 (12.85%)^&^
Lumbago	29 (43.28%)^*^	14 (20.89%)^*#^	10 (14.92%)	10 (14.28%)	4 (5.71%)	4 (5.71%)
Arthralgia	39 (58.20%)^*^	15 (22.38%)^*#^	11 (16.41%)	18 (25.71%)	7 (10.00%)	5 (7.14%)

Data are given as the number (%). ^*^ *P*<0.05 relative to the Kunming group, ^#^ *P*<0.05, relative to 3^rd^ day values, ^&^ *P*<0.05, relative to 50^th^ day values.

The most common symptoms were fatigue, dizziness and coughing, reported in 73.13%, 73.13%, and 73.13% of 67 subjects on the 3^rd^ day after the return to Chongqing. These symptoms were reported in 50.00%, 50.00%, and 52.85% of 70 subjects on the 3^rd^ day after the return to Kunming.

On the 3^rd^ day, the incidence of all symptoms except fatigue, expectoration, increased appetite, constipation, diarrhea, and abdominal pain was higher among subjects who returned to Chongqing than among those who returned to Kunming. The incidence of all symptoms decreased significantly over time. On the 100^th^ day after the return, unresponsiveness, headache, coughing, and flustering were only slightly higher in the Chongqing group than in the Kunming group.

These data suggest that the greater the descent from high altitude to low, the greater the incidence of symptoms of altitude de-acclimatization syndrome.

### Changes in pH, PaO_2_, PaCO_2_, and SaO_2_ after the return

In the low altitude, the samples were collected successfully. In the high altitude, the samples collection rate is 94.7% (36/38).

No significant differences in pH were observed among values recorded at baseline, high altitudes, and on the 3^rd^ day after the return to Chongqing or Kunming. PaO_2_ and SaO_2_ values were higher on the 3^rd^ day than at high altitudes. Values recorded at high altitudes showed no difference from baseline values. The levels of PaCO_2_ were higher on the 3^rd^ day after the return to Chongqing than at high altitudes. No other differences were observed within these Kunming groups. Compared with control group at high altitudes 6 months time point, PaO_2_, PaCO_2_ and SaO_2_ were significant elevated in HDAS group. (Tables 4 and 5).

**Table 4:** pH, PaO2, PaCO2, and SaO2 of subjects after returning to Chongqing.

	HDAS group	Control group
**Baseline**	**n = 23**	**n = 25**
pH	7.39±0.01	7.39±0.03
PaO_2_ (hPa)	126.97±5.59	128.37±5.26
PaCO_2_ (hPa)	49.38±2.90	49.46±2.52
SaO_2_ (%)	97.36±0.53	98.36±0.46
**High altitudes 6 months**	**n = 16**	**n = 24**
pH	7.45±0.06	7.41±0.03
PaO_2_ (hPa)	81.58±4.33^*△^	129.12±4.22
PaCO_2_ (hPa)	35.00±4.41^*△^	51.28±3.27
SaO_2_ (%)	87.32±1.45^*△^	97.85±0.89
**3^rd^ day**	**n = 13**	**n = 22**
pH	7.40±0.03	7.38±0.04
PaO_2_ (hPa)	125.84±3.27^&^	128.78±4.38
PaCO_2_ (hPa)	46.85±1.94^&^	51.79±3.83
SaO_2_ (%)	96.78±0.85^&^	97.45±0.63

Data are given as the mean ± SD. ^*^ *P*<0.05, relative to baseline, ^&^ *P*<0.05, relative to high altitude values recorded at 6 months, ^△^ *P*<0.05, relative to the control group

**Table 5:** pH, PaO2, PaCO2, and SaO2 of subjects after returning to Kunming.

	HDAS group	Control group
**Baseline**	**n = 31**	**n = 33**
pH	7.40±0.02	7.42±0.04
PaO_2_ (hPa)	119.76±6.49	119.49±5.26
PaCO_2_ (hPa)	45.39±2.75	46.51±3.52
SaO_2_ (%)	95.36±0.83	96.11±0.59
**High altitudes 6 months**	**n = 20**	**n = 27**
pH	7.43±0.09	7.39±0.05
PaO_2_ (hPa)	88.87±4.33^*△^	121.12±6.22
PaCO_2_ (hPa)	37.45±5.47^△^	47.32±2.27
SaO_2_ (%)	89.32±1.32^*△^	94.85±1.23
**3^rd^ day**	**n = 16**	**n = 22**
pH	7.41±0.07	7.40±0.06
PaO_2_ (hPa)	120.10±7.48^&^	12.35±4.38
PaCO_2_ (hPa)	42.73±4.13	44.78±3.98
SaO_2_ (%)	94.42±1.02^&^	95.25±0.98

Data are given as the mean ± SD. ^*^ *P*<0.05, relative to baseline, ^&^ *P*<0.05, relative to high altitude values, ^△^ *P*<0.05, relative to the control group

### Changes in hematologic parameters after the return

Changes in hematologic parameters of subjects on the 3^rd^, 50^th^, and 100^th^ days after the return to Chongqing and Kunming after 6 months of exposure to high altitudes are illustrated in detail in Tables 6 and 7.

**Table 6:** Whole blood test results of subjects after returning to Chongqing.

Group	Parameter	Baseline	High altitudes 6 months	3^rd^ day	50^th^ day	100^th^ day
HDAS group (n = 67).	RBC (×10^12^)	4.90±0.23	5.89±0.57^*△^	5.43±0.62^*&△^	4.97±0.38^&#^	4.91±0.30^&#^
	Hb (g/L)	143.70±11.14	177.94±13.02^*△^	168.36±9.32^*&△^	156.85±8.87^&#△^	146.73±8.45^&#^
	Hct (%)	40.19±2.48	55.69±4.03^*△^	47.11±3.02^*&△^	41.46±2.71^&^	42.79±2.54^&^
	MCV (fL)	81.96±3.58	93.29±6.52^*△^	91.72±5.72^*△^	90.64±5.51^*^	85.27±4.26^&#^^
	MCH (pg)	31.35±1.70	30.36±2.51	32.68±2.69^*&△^	31.23±2.09^&#^	32.02±1.21^&#^
	RDW (%)	13.96±0.46	13.97±0.92	13.61±0.98^&^	11.76±0.77^*&#△^	13.62±0.48̂^△^
	WBC (×10^9^)	5.84±0.96	6.07±1.64	7.47±1.63^*&△^	5.90±1.85^#^	5.61±1.22^#^
	Neu (%)	50.99±6.86	53.71±8.67	63.09±6.41^*&△^	54.24±8.35^#^	54.49±7.17^#^
Control group (n = 92)	RBC (×10^12^)	4.85±0.37	4.97±0.29	4.79±0.34	4.80±0.16	4.90±0.18
	Hb (g/L)	143.53±6.92	149.26±7.15	147.40±6.44	147.33±6.12	145.26±6.61
	Hct (%)	41.74±2.92	40.97±3.38	41.97±2.93	41.52±3.34	40.70±3.06
	MCV (fL)	83.96±3.17	84.74±4.33	85.16±3.73	85.62±3.47	84.70±3.05
	MCH (pg)	30.25±1.99	29.30±1.66	29.98±1.53	30.08±1.44	30.19±1.30
	RDW (%)	14.09±0.39	13.95±0.60	13.85±0.71	14.08±0.77	13.99±0.68
	WBC (×10^9^)	5.98±0.98	5.75±0.86	5.81±0.73	5.90±0.91	5.87±0.79
	Neu (%)	53.58±6.18	55.72±3.24	55.46±3.34	56.77±3.31	56.48±3.52

Data are given as the mean ± SD. RBC  =  red blood cell, Hb  =  hemoglobin, Hct  =  hematocrit, MCV  =  mean corpuscular volume, MCH  =  mean corpuscular hemoglobin, MCHC  =  mean corpuscular hemoglobin concentration, RDW  =  red blood cell volume distribution width, WBC  =  white blood cell, Neu  =  neutrophil. ^*^ *P*<0.05, relative to baseline, ^&^ *P*<0.05, relative to high altitude values, ^#^ *P*<0.05, relative to 3^rd^ day values, ^*P*<0.05, relative to 50^th^ day values, ^△^ *P*<0.05, relative to the control group.

**Table 7:** Whole blood test results of subjects after returning to Kunming.

Group	Parameter	Baseline	High altitudes 6 months	3^rd^ day	50^th^ day	100^th^ day
HDAS group (n = 70)	RBC (×10^12^)	5.15±0.43	5.69±0.32^*△^	5.54 ±0.50^*&△^	5.23±0.31^&#^	5.08±0.38^&#^
	Hb (g/L)	151.65±9.31	180.50±15.90^*△^	169.43±11.12^*&△^	157.27±8.51^&#^	149.85±8.90^&#^
	Hct (%)	43.42±2.71	57.89±4.16^*△^	49.13±3.10^*&△^	44.18±3.30^&^	43.92±2.25^&^
	MCV (fL)	89.07±4.02	95.59±12.14^*^	88.82±5.49^&^	90.30±5.16^&^	90.56±4.37^&^
	MCH (pg)	30.48±1.79	35.58±3.14^*△^	32.00±2.92^*&△^	32.70±2.35^*&△^	31.76±1.92^*&△^
	RDW (%)	13.48±0.81	14.14±1.70	12.91±0.58^*&△^	13.00±0.93^*&^	13.08±0.69^*&^
	WBC (×10^9^)	5.47±1.07	6.73±0.58^△^	7.14±0.46^*&△^	5.64±0.78^&#^	5.22±0.93^&#^
	Neu (%)	52.89±3.27	56.80±3.53	65.80±3.27^*△^	54.60±6.36^#^	53.77±7.76^#^
Control group (n = 87)	RBC (×10^12^)	5.12±0.24	5.19±0.55	5.18±0.15	5.13±0.30	5.09±0.24
	Hb (g/L)	153.40±7.92	154.94±6.08	151.84±9.13	154.75±4.31	150.68±9.07
	Hct (%)	43.34±2.56	42.48±2.87	43.46±2.43	43.44±2.77	42.90±2.29
	MCV (fL)	88.95±2.58	90.18±2.76	88.60±2.93	89.45±2.88	89.33±3.03
	MCH (pg)	30.16±2.09	31.12±1.28	29.73±2.01	30.30±2.17	30.55±1.39
	RDW (%)	13.46±0.91	13.36±0.70	13.41±0.65	13.48±1.00	13.32±0.80
	WBC (×10^9^)	5.30±1.06	5.53±1.07	5.50±1.02	5.51±1.09	5.52±1.00
	Neu (%)	55.36±8.81	55.78±4.04	57.63±5.46	56.63±5.58	57.14±4.05

Data are given as the mean ± SD. RBC  =  red blood cell, Hb  =  hemoglobin, Hct  =  hematocrit, MCV  =  mean corpuscular volume, MCH  =  mean corpuscular hemoglobin, MCHC  =  mean corpuscular hemoglobin concentration, RDW  =  red blood cell volume distribution width, WBC  =  white blood cell, Neu  =  neutrophil. ^*^ *P*<0.05, relative to baseline, ^&^ *P*<0.05, relative to high altitude values, ^#^ *P*<0.05, relative to 3^rd^ day values, ^*P*<0.05, relative to 50^th^ day values, ^△^ *P*<0.05, relative to the control group.

RBC counts were lower on the 3^rd^ day after the return than at high altitudes but still higher than baseline values (both *P*<0.05). They were lower on the 50^th^ and 100^th^ days than on the 3^rd^ day (all *P*<0.05). Values had returned to baseline by the 50^th^ day in the Chongqing group and by the 100^th^ day in the Kunming group (*P*>0.05).

Hb levels were lower on the 3^rd^ day after the return than those recorded at high altitudes (*P*<0.05). The levels recorded on the 50^th^ and 100^th^ days were lower than those recorded on the 3^rd^ day (both *P*<0.05). Hct levels were significantly lower on the 3^rd^ day after the return than at high altitudes. The levels of Hb and Hct had returned to near-baseline levels by the 100^th^ day (*P*>0.05).

Compared with control group, RBC counts, Hb and Hct were higher on the High altitude 6 months and 3^rd^ day after the return in HDAS group (*P*<0.05). The levels of Hb were found to be significantly higher on the 50^th^ days than control group in Chongqing (*P*<0.05).

However, on the 3^rd^ day after the return, WBC counts were significantly higher than at baseline or at high altitudes (both *P*<0.05). WBC counts were significantly lower on the 50^th^ and 100^th^ days than on the 3^rd^ (*P*<0.05). No statistically significant differences were observed between the 50^th^ and 100^th^ days (*P*>0.05). The relative number of neutrophils showed similar tendencies.

**Figure 3:**
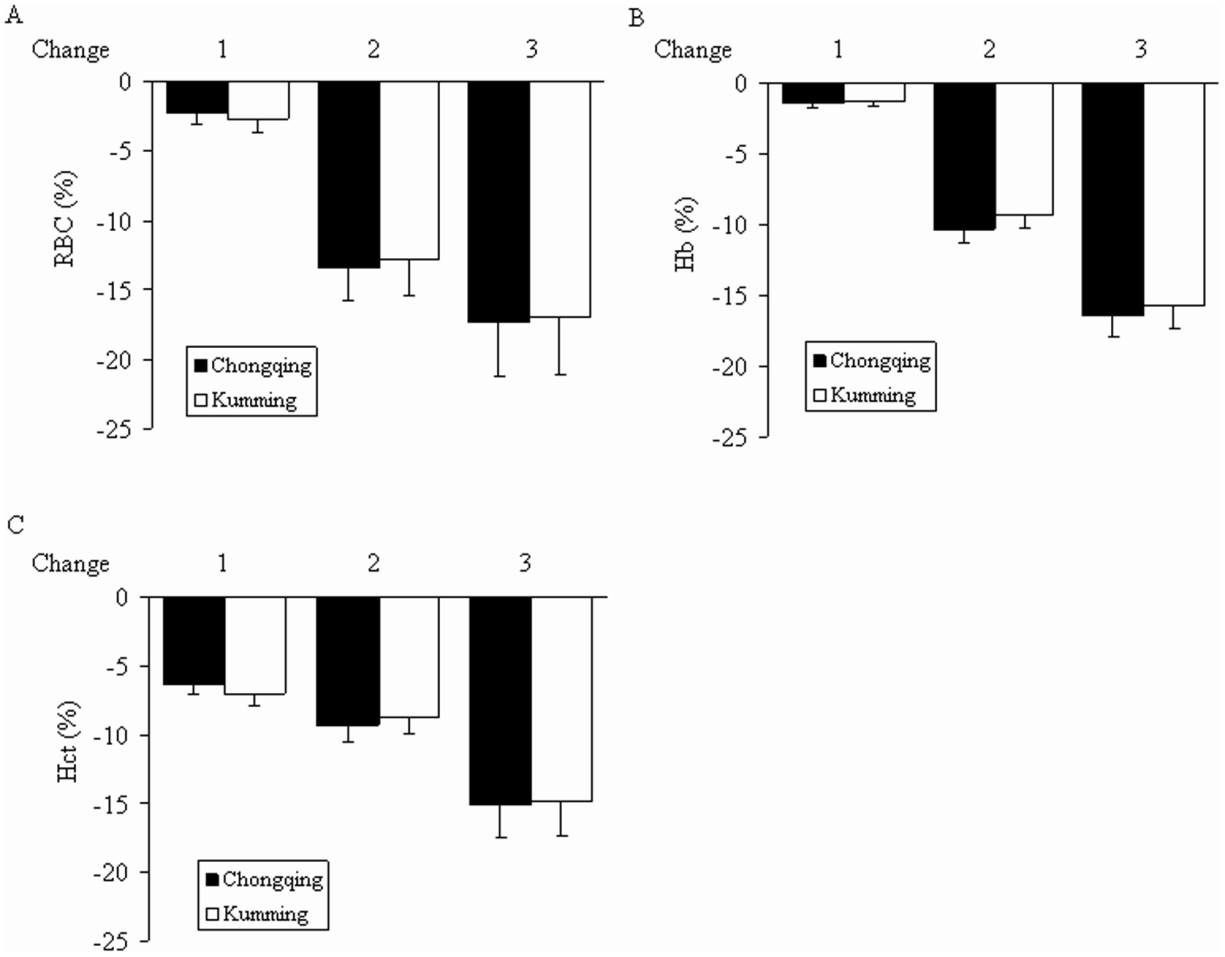
Rates of change of RBC, Hb, and Hct. Data are presented as mean ± SD. The rate of change of (A) RBC, (B) Hb, and (C) Hct. Values recorded in subjects at high altitudes served as a baseline. Rate of change 1 = (value on the 3rd day-value at high altitudes)/value at high altitudes×100%; rate of change 2 = (value on the 50th day-value at high altitudes)/value at high altitudes×100%, rate of change 3 = (value on the 100th day-value at high altitudes)/value at high altitudes×100%. Rates of change 1, 2, and 3 showed no significant differences between the Chongqing and Kunming groups (*P*>0.05).

Rate of change in symptom scores (1 and 2) groups were not significantly different between the Chongqing and Kunming groups (*P*>0.05). Hb and Hct showed similar tendencies in the two groups (Figure 3).

### Changes in CK, CK-MB, and LDH levels after the return

Changes in CK, CK-MB, and LDH of subjects on the 3^rd^, 50^th^, and 100^th^ days after the return to Chongqing and Kunming are illustrated in detail in Tables 8 and 9.

**Table 8:** Serum CK, CK-MB, and LDH of subjects after returning to Chongqing.

Group	Parameter	Baseline	High altitudes 6 months	3^rd^ day	50^th^ day	100^th^ day
HDAS group (n = 67)	CK (IU/L)	88.70±17.77	92.90±21.80	121.73±28.91^*&△^	97.57±21.91^#^	94.02±18.20^#^
	CK-MB (IU/L)	16.30±2.62	16.50±3.59	21.91±3.18^*&△^	17.63±3.23^#^	15.80±4.61^#^
	LDH (IU/L)	147.06±22.28	151.98±34.40	200.33±26.06^*&△^	177.18±31.87^*&#△^	146.38±22.52^#^^
Control group (n = 92)	CK (IU/L)	87.50±16.09	91.37±17.95	88.62±12.11	91.50±18.64	89.87±11.36
	CK-MB (IU/L)	15.95±2.20	14.76±2.25	16.33±1.69	15.74±1.91	14.92±2.49
	LDH (IU/L)	146.53±19.15	151.16±16.95	142.64±17.57	152.69±18.17	149.14±21.56

Data are given as the mean ± SD. CK  =  creatine kinase, CK-MB  =  creatine kinase–MB, LDH  =  lactate dehydrogenase. ^*^ *P*<0.05, relative to baseline, ^&^ *P*<0.05, relative to high altitude values, ^#^ *P*<0.05, relative to 3^rd^ day values, ^*P*<0.05, relative to 50^th^ day values, ^△^ *P*<0.05, relative to the control group.

**Table 9:** Serum CK, CK-MB, and LDH of subjects after returning to Kunming.

Group	Parameter	Baseline	High altitudes 6 months	3^rd^ day	50^th^ day	100^th^ day
HDAS group (n = 70)	CK (IU/L)	88.52±14.30	90.63±20.70	119.00±15.42^*&△^	96.36±18.96^#^	90.36±14.37^#^
	CK-MB (IU/L)	15.04±1.38	16.07±3.16	21.41±3.26^*&△^	17.12±3.03^#△^	16.39±3.33^#^
	LDH (IU/L)	150.79±20.99	153.44±26.42	198.02±35.55^*&△^	176.41±26.94^*&#△^	149.10±15.29^#^^
Control group (n = 87)	CK (IU/L)	90.96±21.89	88.19±21.06	90.74±18.24	86.03±14.74	93.15±15.55
	CK-MB (IU/L)	15.09±2.48	15.50±2.35	15.71±3.36	15.53±2.94	14.90±3.04
	LDH (IU/L)	148.72±17.78	150.16±20.56	147.29±18.50	152.73±18.93	150.75±19.20

Data are given as the mean ± SD. CK  =  creatine kinase, CK-MB  =  creatine kinase–MB, LDH  =  lactate dehydrogenase. ^*^ *P*<0.05, relative to baseline, ^&^ *P*<0.05, relative to high altitude values, ^#^ *P*<0.05, relative to 3^rd^ day values, ^*P*<0.05, relative to 50^th^ day values, ^△^ *P*<0.05, relative to the control group.

The levels of CK and CK-MB were significantly higher on the 3^rd^ day after the return than at baseline or at high altitudes (*P*<0.05). Values were lower on the 50^th^ and 100^th^ day than on the 3^rd^ day (*P*<0.05). No significant differences in CK, CK-MB, or LDH were observed between baseline values and those recorded at high altitudes.

The levels of LDH were found to be significantly higher on the 3^rd^ and 50^th^ days than at baseline and or at high altitudes (*P*<0.05). Their values were significantly lower, approaching baseline, on the 100^th^ day than on the 3^rd^ and 50^th^ days (*P*<0.05).

Compared with control group, CK and CK-MB were higher on the High altitude 6 months and 3^rd^ day after the return in HDAS group (both *P*<0.05). The levels of CK-MB were found to be significantly higher on the 50^th^ days than control group in Kunming (*P*<0.05). The levels of LDH were found to be significantly higher on the high altitudes 6 months, 3^rd^ and 50^th^ days in HDAS group than in control group (*P*<0.05).

**Figure 4:**
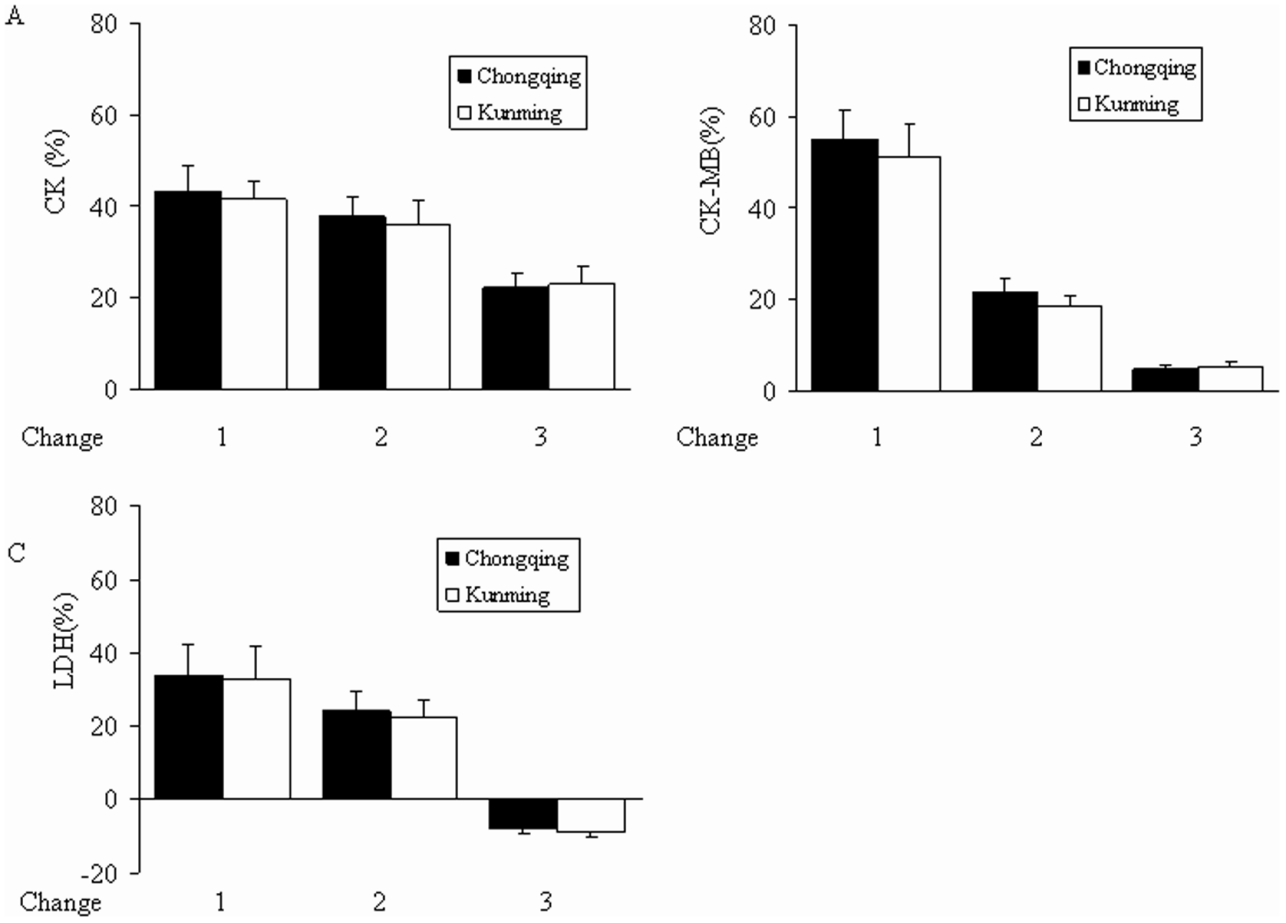
Rates of change of CK, CK-MB, and LDH. Data are presented as mean ± SD. The rate of change rate of (A) CK, (B) CK-MB, (C) LDH. Values recorded in subjects at high altitudes served as the baseline. Rate of change 1 = (value on 3rd day-value at high altitudes)/value at high altitudes×100%; rate of change 2 = (value on 50th day-value at high altitudes)/value at high altitudes×100%; rate of change 3 = (value at 100th day-value at high altitudes)/value at high altitudes×100%. Rates of change 1, 2, and 3 were not significantly different between the Chongqing group and Kunming group (*P*>0.05).

Rates of change rate 1, 2, and 3 were not significantly different between the Chongqing and Kunming groups (*P*>0.05). CK-MB and LDH showed similar tendencies (Figure 4).

## Discussion

More studies of high altitude de-acclimatization syndrome of subjects who visit high-altitude areas are necessary. Improvements in the transportation system in Tibet and Qinghai, the development of tourism and resources in these areas, and improvements in the conditions for the construction of manufacturing facilities have increased the number of people who come to these high-altitude plateau areas for work and travel, usually for several months or one year, after which they return home. These visitors come from all over the world, not only from within China. These temporary residents typically show the following two distinct features. The first is that they tend to be adults between 15 and 70 years old. Among working populations, the majority are young and middle-aged (20–50 years old). The other is that they typically remain at high altitudes for only short periods of time (≤1 year). This includes people who work at high altitudes for half a year to one year and then return to low-altitude areas. There have been studies on high-altitude de-acclimatization syndrome and relevant diagnostic criteria, but most of them have focused on individuals who lived at high altitudes for long periods of time (over 5 years, up to 20 years) [16], [20]. Current studies on individuals who return to low altitude areas after fixed time exposure to high altitudes are still rare.

The establishment of diagnostic criteria for high-altitude de-acclimatization syndrome after fixed time exposure to high altitudes is crucial to prevention and treatment. The present study focused on symptoms of high-altitude de-acclimatization among 136 subjects who worked in the Tibetan plateau (≥3600 m) about half a year between 2009 and 2010 and returned to Chongqing and Kunming afterwards. It also discusses diagnostic criteria suitable for use with high-altitude de-acclimatization after a fixed time (≤1 year) of exposure to high altitudes. Our epidemiological data showed that, upon returning to lowlands or plains, subjects exhibited the following symptoms: fatigue, sleepiness, insomnia, unresponsiveness, memory loss, fidgetiness, headache, throat pain or discomfort, coughing, expectoration, chest tightness, flustering, increased appetite, decreased appetite, constipation, diarrhea, abdominal distention, abdominal pain, lumbago, and arthralgia. Other symptoms of discomfort typically found in long-term visitors to high altitudes upon their return to lowland areas, such as numbness in all acroanesthesia, cardiodynia, slowed pulse, lower extremity edema, facial swelling, and cyanosis, were not observed. Absence of these symptoms in high-altitude visitors might be due to the fact that the damage to their hearts and lungs that occurred after their return to lowland areas was not severe. The duration and severity of the influences of environmental factors such as hypoxia and cold are here proposed as modified diagnostic criteria for a fixed time de-acclimatization syndrome. This may facilitate understanding, diagnosis, and treatment of high-altitude de-acclimatization among people who visit high-altitude areas for fixed times of time, benefiting the physical health, mental health, and quality of life of individuals who temporary work on the Tibetan plateau and then return to lowland plains. It may also support further in-depth investigations of the pathogenesis of high-altitude de-acclimatization after exposure to high-altitude environments.

Evaluation of the de-acclimatization syndrome is key to establishing diagnostic criteria. Symptoms of 136 subjects who returned to low altitude upon exposure of high altitudes 6 months were evaluated in this project. Until the 3rd day after each person's return, the symptoms of high-altitude de-acclimatization remained steady. Both symptom scores and incidence of each symptom decreased on the 50^th^ day, and then decreased further on the 100^th^ day. We speculate that it may be associated with the attenuation of damage from low-altitude re-acclimazation and reoxygenation. In previous study, we surveyed personnel who performed earthquake relief work at Yushu in Qinhai and followed up with them for 120 days after they returned to lowland plains areas. These individuals showed symptoms of discomfort, including dizziness, fatigue, sleepiness, and other symptoms, and the rate of occurrence was as high as 100% [23]. Studies conducted in other countries have shown that mountain climbers returning to lowland areas also show some symptoms of de-acclimatization, in this case called “oxygen intoxication syndrome.” Specifically, they feel top-heavy and sleepy, and they fall asleep easily and become hard to wake up, as if they were drunk. The present study showed that, upon exposure to the lowland O_2_ levels, which can reach 21%, reoxygenation proceeded, and the blood oxygen level increased by 6–8% after the return. Other studies have shown that, after high-altitude visitors return to lowlands; their red blood cells remain capable of carrying high amounts of oxygen, which leads to increased blood oxygen content and pleonastic blood [14]. This causes reoxygenation of the body after hypoxia, which can induce the activation of many signal pathways in neuronal cells, myocardial cells, endothelial cells, and small intestine tissues and induce apoptosis and damage to these cells [22], [24]–[30]. We believe that the occurrence of high-altitude de-acclimatization is closely related to reoxygenation. Some believe that the damage to organs, such as the brain, observed after the subjects returned to lowland plains were a continuation of damage caused by hypoxia at high altitudes [31]–[33]. However, studies on populations with high-altitude de-acclimatization conducted by our group and by Cui's group have shown that none of the symptoms of de-acclimatization are observable while the subjects are still in the high-altitude environment [15], [16]. This suggested that high-altitude de-acclimatization syndrome may not be directly related to hypoxia-induced damage. The results of the present study also showed that the overall symptom scores of subjects returning to Kunming were lower than those returning to Chongqing at all points in time. On the 3rd day after their return, the rate of occurrence of each symptom among subjects returning to Kunming was also significantly lower than among subjects returning to Chongqing. This suggests that the severity of high-altitude de-acclimatization and the rate of occurrence of related symptoms may be correlated with the difference in altitude between the high-altitude area and the area to which the subjects returned.

In addition, we observed that physiological condition of 41 subjects (21 subjects from Chongqing and 20 from Kunming) after returned to low altitude from 1st day to 1 week. Immediately upon returning to low-altitude areas, subjects felt excited and comfortable, breathed smoothly, and enjoyed good appetite. Few subjects complained of fatigue. The next day, many people began to show some symptoms, including fatigue, headache, dizziness, sleepiness, insomnia, unresponsiveness, memory loss, agitation, sore throat, coughing, expectoration, chest tightness, increased appetite, reduced appetite, abdominal distension, diarrhea, and unexplained joint pain. On the third day, most subjects' symptoms were worse than on the 2^nd^ day. The symptom scores had not changed significantly by the 5^th^ and 7^th^ days compared to the 3^rd^ day. Indeed, few subjects' symptoms improved and disappeared on 3^rd^ day.

Hematological testing is an indispensable auxiliary tool in the diagnosis of high-altitude de-acclimatization syndrome. The present study showed that on 3^rd^ day after the subjects' return to lowlands, the subjects' RBC, Hb, and Hct levels were significantly lower than they had been at high altitudes but still higher than baseline. On the 50th day after the return, the values of these indices were no longer different from baseline.Hypoxia causes RBC, Hb, and Hct levels to increase significantly after individuals ascend to high altitudes. They stabilize at relatively high levels [14]. When these individuals return to lowlands, RBC, Hb, and Hct all gradually returned to baseline or even dropped below baseline [34], [35]. However, the time required for these indices to return to baseline may vary due to differences in the duration of exposure to high altitudes, the altitude of the plateau area, and the intensity of labor performed at high altitudes. Hb and Hct levels might recover in 1 day or take as long as 61 days [3], [35]–[38]. We speculate that it may be associated with that re-expansion of plasma volume to reverse the hemoconcentration, prevention of recruitment of new erythrocytes by down-regulating erythropoiesis and destruction of excess erythrocytes, recycling of Hb, and re-uptake of erythrocytes by the spleen.

Myocardial zymography is an important parameter in the evaluation of damage to the heart muscles of subjects with high-altitude de-acclimatization syndrome. The results of the present study showed that, in both studied populations, the CK, CK-MB, and LDH levels did not differ significantly from baseline after 6 months of exposure to high altitudes. On the 3^rd^ day after the return to lowland areas, the CK, CK-MB, and LDH levels were significantly higher than baseline. On the 50^th^ day, CK and CK-MB levels were not significantly different from baseline. On the 100^th^ day, the LDH levels were not significantly different from baseline. These data suggest that reoxygenation activates multiple pathways that induced the damage of the heart muscles [27]. This increase the expression of CK, CK-MB, and LDH in the plasma, as the body transitions from a hypoxic state to a reoxygenated one. On the other hand, parameters become gradually normalised, myocardial damage was improvement because of sea level re-acclimatization.

The rates of improvement in the subjects' symptoms and the rates of change in RBC, Hb, Hct, CK, CK-MB, and LDH levels were not significantly different between individuals who returned to Kunming and those who returned to Chongqing, suggesting that the rate of recovery may not be related to the altitude of the area to which the sbujects returned. The underlying cause of this remains unclear. However, neutrophil granulocytes were found to play an important role in the non-specific cellular immune system of the blood. On the 3^rd^ day after the return, the relative white blood cell and neutrophil granulocyte counts were significantly higher than baseline. This may be related to hypoxia/reoxygenation-induced tissue damage and the release of inflammatory mediators [39].

Our study has some limitations. (1) It was not a randomized multicenter study. (2) The regions to which the subjects returned included only two areas and these areas are not representative of all visitors to the plateau. (3) The subjects' ages were only representative of some visitors. (4) All subjects were men and most of them were from 25 to 45 years old. (5) Several factors could not be controlled well between Lhasa and low-altitude areas. For instance, the time zone is different, the types of meat the subjects ate were different, and different rest-time activities were available at Lhasa than at low altitudes. (6) The present study is a preliminary study and it requires only a basic framework for the diagnosis of high-altitude de-acclimatization syndrome conditions.

## Conclusions

In conclusion, in the present study, we established diagnostic criteria and symptom evaluation criterion for high-altitude de-acclimatization in individuals returning to lowlands after exposure to high altitudes lasting less than 1 year. We evaluated the associations between symptom scores and differences in altitude, symptom characteristics, hematological cell levels, blood-gas levels, and myocardial enzyme levels. All values slowly returned to baseline. However, recovery from de-acclimatization syndrome was found to take a long time, more than 100 days.
