# Correction: Analysis of High-Altitude De-Acclimatization Syndrome after Exposure to High Altitudes: A Cluster-Randomized Controlled Trial

**DOI:** 10.1371/annotation/6c085b4d-9f37-4d48-84c9-5abfd691d2c7

**Published:** 2013-07-18

**Authors:** Binfeng He, Jianchun Wang, Guisheng Qian, Mingdong Hu, Xinming Qu, Zhenghua Wei, Jin Li, Yan Chen, Huaping Chen, Qiquan Zhou, Guansong Wang

The number of the third listed grant from the National Science Foundation of China is incorrect. The correct grant number is: 81170067. 

